# Hypotheses and tracking results about the longest migration: The case of the arctic tern

**DOI:** 10.1002/ece3.5459

**Published:** 2019-08-02

**Authors:** Thomas Alerstam, Johan Bäckman, Johanna Grönroos, Patrik Olofsson, Roine Strandberg

**Affiliations:** ^1^ Department of Biology Lund University Lund Sweden; ^2^ Faculty of Natural Sciences Kristianstad University Kristianstad Sweden; ^3^ Heberg, Sweden

**Keywords:** Antarctica, arctic tern, bird migration, global migration, population segregation

## Abstract

The arctic tern *Sterna paradisaea* completes the longest known annual return migration on Earth, traveling between breeding sites in the northern arctic and temperate regions and survival/molt areas in the Antarctic pack‐ice zone. Salomonsen (1967, *Biologiske Meddelelser, Copenhagen Danske Videnskabernes Selskab*, **24**, 1) put forward a hypothetical comprehensive interpretation of this global migration pattern, suggesting food distribution, wind patterns, sea ice distribution, and molt habits as key ecological and evolutionary determinants. We used light‐level geolocators to record 12 annual journeys by eight individuals of arctic terns breeding in the Baltic Sea. Migration cycles were evaluated in light of Salomonsen's hypotheses and compared with results from geolocator studies of arctic tern populations from Greenland, Netherlands, and Alaska. The Baltic terns completed a 50,000 km annual migration circuit, exploiting ocean regions of high productivity in the North Atlantic, Benguela Current, and the Indian Ocean between southern Africa and Australia (sometimes including the Tasman Sea). They arrived about 1 November in the Antarctic zone at far easterly longitudes (in one case even at the Ross Sea) subsequently moving westward across 120–220 degrees of longitude toward the Weddell Sea region. They departed from here in mid‐March on a fast spring migration up the Atlantic Ocean. The geolocator data revealed unexpected segregation in time and space between tern populations in the same flyway. Terns from the Baltic and Netherlands traveled earlier and to significantly more easterly longitudes in the Indian Ocean and Antarctic zone than terns from Greenland. We suggest an adaptive explanation for this pattern. The global migration system of the arctic tern offers an extraordinary possibility to understand adaptive values and constraints in complex pelagic life cycles, as determined by environmental conditions (marine productivity, wind patterns, low‐pressure trajectories, pack‐ice distribution), inherent factors (flight performance, molt, flocking), and effects of predation/piracy and competition.

## 1 INTRODUCTION

The arctic tern has a very special reputation among migratory birds since more than a 100 years, being appointed by Cooke (1915) as the “world's migration champion.” At that time, the arctic tern had recently been encountered during ship expeditions to the Southern Ocean, notably the Weddell Sea region. The migration routes by which the terns completed their amazing journeys between the polar regions were completely unknown, as also admitted by Cooke (1915).

During the first half of the 20th century, ringing recoveries of arctic terns accumulated. So did field observations of migrating terns along the coasts and over the open ocean as reported from ship journeys (e.g., Austin, 1928, Wynne‐Edwards, 1935). With all observations combined, a distinct pattern of global migration routes started to emerge, as reconstructed by Kullenberg (1946). Kullenberg (1946) pointed out the existence of two main flyways for the arctic terns on their southward migration. A major flyway runs in the East Atlantic Ocean from European waters and continues along the African coast toward South Africa, from where the terns cross to the Weddell Sea. Terns from northeastern North America and Greenland travel eastward across the North Atlantic to connect with this flyway, and terns from West Siberia and northern Europe connect by westerly flights from their breeding sites. A second main flyway is in the eastern Pacific Ocean, running from Alaska along the coasts of North and South America. This flyway is presumably used by arctic terns breeding in eastern Siberia and in Alaska (the locations of the migratory divides between the terns using the two main flyways still remain elusive).

However, forthcoming reports about the winter distribution and migration passages of arctic terns could not be fully reconciled with the migration pattern suggested by Kullenberg (1946). Even if the highest winter densities of arctic terns have been reported from the pack ice in the Weddell Sea, the arctic terns seemed to have a more circumpolar winter distribution associated with the pack ice around the whole Antarctic continent. Furthermore, an important southeastward migration passage of arctic terns was reported in November to December from Heard Island (53S, 73E; Downes, 1952), far to the east of South Africa. Observations and ringing recoveries were also reported in October–December from the southern Indian Ocean, southwestern and southern Australia, and New Zealand (Storr, 1958). Based on these new records of arctic terns, Storr (1958) proposed that many terns during autumn migration do not continue southward from South Africa but migrate eastward into the southern Indian Ocean, some continuing as far as southern Australia and New Zealand.

Building on these new observations and suggestions, also summarized by Dorst (1962), Salomonsen (1967) put forward a hypothetical comprehensive interpretation of the migratory movements of the arctic tern in the Southern Ocean. We think that Salomonsen's (1967) seminal synthesis and ideas are still of unique importance for understanding the long‐distance pelagic migration of the arctic tern, and we will use it as the main fundament for this study. He suggested four key factors as the principal ecological and evolutionary determinants for the terns' migration pattern: (1) food distribution, (2) wind patterns (low‐pressure trajectories), (3) pack ice, and (4) molt habits. Salomonsen's hypothesis deals with four main phases of the arctic tern movement in the Southern Ocean: (a) the southward migration from subtropical waters to the Antarctic pack‐ice zone, (b) the terns' situation and movements in this zone during the Antarctic summer (the terns' wintering period), (c) the northward departure from the Antarctic wintering range, and (d) the movements of young terns which do not migrate to the breeding range in their first year. We summarize very briefly the main predictions for these phases or aspects as follows (for more detailed explanations, see Salomonsen, 1967, also available at http://www.royalacademy.dk/Publications/High/267_Salomonsen,%20Finn.pdf):

- *Southward migration to the pack‐ice zone*: When leaving the waters at South Africa (or southern South America), the terns do not head due south because of the risk of uncontrolled drift over the stormy ocean but are adapted to exploit the winds in low‐pressure systems to reach the pack ice in the safest and most efficient way. This means that they follow the main low‐pressure trajectories toward ESE or SE. Terns passing South Africa will mainly follow the Kerguelen low‐pressure trajectory system, reaching the Antarctic pack‐ice zone between longitudes 30E and 150E. A minority of the terns are carried by the strong westerly winds all the way across the southern Indian Ocean to Australia and even New Zealand, and they will utilize low‐pressure trajectories toward pack‐ice regions even further to the east. Arctic terns leaving South America will follow low‐pressure trajectories toward the Weddell Sea.
- *Stay in the pack‐ice zone*: The arctic terns spend the months November–February in the Antarctic pack‐ice zone. During this period, they exploit the rich supply of krill in the upwelling waters. Molting their flight feathers (molt duration about 60 days), they become hampered in their flight and spend much time sitting on the ice. In the early part of this wintering period, the pack‐ice zone extends far north of the Antarctic continent, into the zone of strong westerly winds, making the terns liable to further eastward drift. However, with the gradual melting of the pack ice during the Antarctic summer the wind regime for the terns will change. Strong westerlies will be replaced by polar easterly winds prevailing closer to the Antarctic coastline in the shrinking pack‐ice zone where the terns reside.
- *Departure from Antarctica*: After competing molt, the terns utilize the polar easterly winds and move westward along the Antarctic coast from East Antarctica to the Weddell Sea region. It is from this region that the main exodus from the Antarctic region takes place. This involves terns that have spent months far to the east in the pack‐ice zone as well as terns that have stayed in the Weddell Sea pack ice throughout the main wintering and molt period. The main path of exodus is toward N‐NE toward South Africa and further northward in the Atlantic Ocean.
- *Circumpolar migration by young terns*: Young terns are assumed to be more vulnerable to wind drift than adults during their migration to and stay in the pack‐ice zone. This means that many of them will be carried further eastward to the Pacific sector of Antarctica, from where they will complete a circumpolar migration by continuing eastward to oversummer in the Humboldt or Benguela Currents.

In this study, we used geolocators to track the annual migrations of arctic terns breeding in the Baltic Sea. We analyzed the results to address Salomonsen's predictions and ideas, and to identify the main unsolved questions about the arctic tern migration. Although the geolocator data, because of limited accuracy (Lisovski & Hahn, 2012) and sample size, may not allow strict quantitative statistical tests, these data can still be used for a critical descriptive evaluation of the main ideas. We think that such an evaluation provides an increased understanding of the evolutionary ecology of the arctic tern migration, as will be demonstrated below. In addition, we compared our results with other geolocator studies of arctic terns breeding in Greenland (Egevang et al., 2010), the Netherlands (Fijn, Hiemstra, Phillips, & Winden, 2013), and Alaska (McKnight, Allyn, Duffy, & Irons, 2013) to analyze one further aspect that was not predicted by Salomonsen (1967):

- *Differences in movements and annual cycles between populations using the same flyway*: While Salomonsen (1967) proposed clear differences in the Antarctic distribution between terns using the Atlantic and Pacific flyways, he seemed to have assumed that there was no segregation between different populations of terns within each of these two main flyways. We will discuss the unexpected differences between breeding populations as revealed by the recent geolocator studies.

## 2 METHODS

The fieldwork was carried out 2008–2018 in Landöskärgården, a small archipelago close to the city Kristianstad in southernmost Sweden (56.0°N, 14.5°E). The area hosts between 20 and 45 pairs of arctic terns annually with the majority breeding on small sandy islands. Arctic terns were caught with a nest trap during the incubation period from mid‐May to mid‐July.

We used archival light‐level loggers (“geolocators” hereafter) manufactured by Migrate Technology Ltd (Intigeo^®^ series) and British Antarctic Survey (BAS). The majority of geolocators were mounted on the back with leg harnesses (*n* = 37), while a smaller number were fitted to the tarsus on a plastic ring (*n* = 10). The attached geolocator packages weighed 1.5–2.0 g (including glue, plastic ring, and/or string), that is, <2% of the body mass of the arctic terns, averaging 108 g (*n* = 73) in our study area. Terns equipped with geolocators were also color‐ringed.

We have no indications that geolocators affected the terns in an important way. We regularly observed color‐ringed terns without being able to find their nesting sites (which is difficult in our study area with scattered smaller colonies and solitarily breeding pairs). This had as a consequence that it often lasted more than 1 year (up to 6 years) until birds with geolocators were recaptured. Out of 10 recaptured terns that had been equipped with geolocators, two were recaptured after 1 year, four after 2 years, one after 3 years, two after 5 years, and one after 6 years. This shows that the returning rate is clearly higher than the recapture rate and that terns with geolocators can complete multiple annual cycles in an apparently normal way (but some adverse effects cannot be excluded, cf. Nisbet, Mostello, Veit, Fox, & Afanasyev, 2011).

The geolocators measure and store light intensity in relation to time of day which can be converted into geographical positions, where latitude is determined from solar day/night length and longitude from the time of local noon/midnight (e.g., Ekstrom, 2004). For more details about geolocating, technical data, and processing of light data, see, for example, Fox (2017) and Tøttrup et al. (2012).

We used the IntiProc software (Migrate Technology Ltd; Fox, 2017) including the Geolight package (Lisovski & Hahn, 2012) for converting light into geographical positions. With the software calibration function, we used clean light data from a 10‐day period in the breeding area for calibration of sun angle versus light threshold for obtaining latitude. Comparing with other calibration options (e.g., Hill–Ekstrom calibration), we found no large differences and conclude that breeding area calibration is useful for approximate latitude estimation for a seabird in open environments (with less important shading effects) like the arctic tern. We could not detect any obvious internal clock drift by examining latitudinal and longitudinal graphs, thus making all tracks valid for analyses. Reliable latitude data were missing from a period of about 34 days around autumn as well as spring equinox (missing latitude data from 25 August/8 September to 5/13 October in autumn and from 4/8 March to 5/7 April in spring for the different individuals and journeys). However, longitude data were available throughout these equinox periods. We have used longitude data for our critical comparisons since longitude is based on the time shift of local noon/midnight and thus available during equinox periods and, in addition, more robust and precise than latitude estimation which depends on the sun angle versus light threshold calibration (Lisovski et al., 2012). Light data were carefully inspected for each day to make sure that light changes were correctly assigned to sunrise/sunset for estimation of location (and such assignments were corrected if needed). Location data were omitted if light changes associated with sunrise/sunset could not be reliably determined.

From November, at the terns' arrival to the Antarctic area, to March, when they departed on northward migration, the terns were often exposed to very long polar day lengths without dark night hours. We selected provisional and biased “sunsets” and “sunrises” from the light curve using a higher threshold value than during the rest of the year cycle (standard threshold value = 2). We used threshold values ≥40 for BAS geolocators and ≥100 for Intigeo geolocators. Some birds even experienced full light for several days/weeks during the period of stay in Antarctica, from which no location data could be obtained. The underestimated day lengths during the Antarctic period resulted in inaccurate latitudinal values being biased to an unknown degree toward the north. Still, these estimates allowed us to infer that latitude was about or further south than 60S, which was sufficient for our present purposes (see Results and Discussion). Estimated longitudes will be valid and illustrate the east–west directed movements within the Antarctic zone during the wintering period. The problems and potential solutions of light‐level geolocation in polar regions with high‐light regimes have been treated by Lisovski (2018).

We based our analyses on average values of the two daily positions (midday/midnight) calculated from logger data, as this reduces some of the variation in location estimates as well as the bias in latitude caused by longitudinal movement. Longitude data are most reliable and unaffected by the great uncertainty of latitude estimates during periods around the autumn and spring equinoxes and during the Antarctic summer. Our critical analyses and comparisons with results for other populations have been primarily based on robust longitudinal data. Light‐level as well as location data are available from the Dryad Repository.

## 3 RESULTS

In this Results section, we present and evaluate the timing and course of migration of the Baltic terns based on the geolocator data, dividing the presentation into the period of autumn (southward) migration, the period in the Antarctic zone (south of 60S), and period of spring (northward) migration. In addition, we compare migration patterns during these periods between the different populations of arctic terns that have been tracked using geolocators. We then proceed in the Discussion section to evaluate the observed migration patterns in relation to Salomonsen's (1967) synthesis and hypotheses.

Geolocator data were obtained from eight individuals during 12 annual journeys in the years 2008–2015 (out of 10 recaptured arctic terns, we retrieved data from eight; one geolocator had been dropped from ring and one disappeared during postal service). Four of the individuals were tracked during two consecutive annual migrations. Complete data coverage up to and including the return to the breeding region was obtained for four annual migrations, while data coverage was interrupted or fragmented during the remaining journeys.

**Figure 1:**
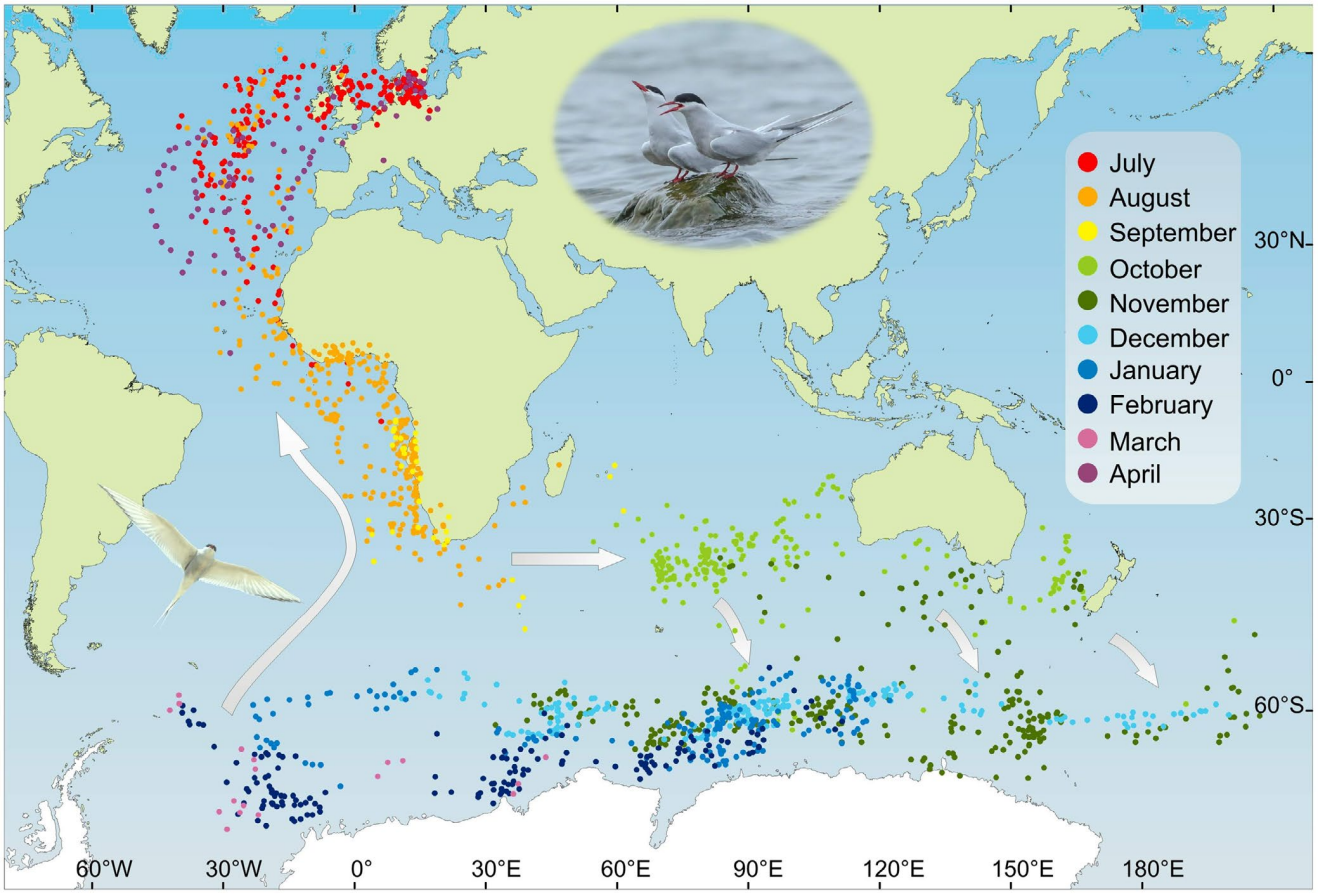
Map (Mercator projection) showing estimated locations of arctic terns from the Baltic Sea during their annual migration cycle. Daily locations based on geolocator data for 12 annual journeys by eight individuals are shown for all journeys combined to illustrate the overall global migration pattern for the study population. Locations are missing due to uncertain and invalid latitude data during periods around the autumn and spring equinoxes. Arrows indicate the most likely movement patterns based on longitude data (Figure 2) during the equinox periods, eastward into the Indian Ocean around the autumn equinox and toward northeast (first) and northwest (later) from the Weddell Sea up the southern Atlantic Ocean around the spring equinox. Small arrows indicate southeastward crossing of the Antarctic Convergence in the longitudinal sector from 80E and eastward (see text). Locations have been provisionally plotted during the terns' wintering period in the Antarctic in spite of the fact that latitude could not be properly estimated but only inferred to be south of 60S during the southern polar summer (see methods about latitude bias for these locations). Maps showing estimated locations for each journey are presented in Appendix 1. The Mercator projection is conformal and true to compass direction. However, it is not true to distance and area, with polar regions greatly exaggerated in size (Gudmundsson & Alerstam, 1998)

**Figure 2:**
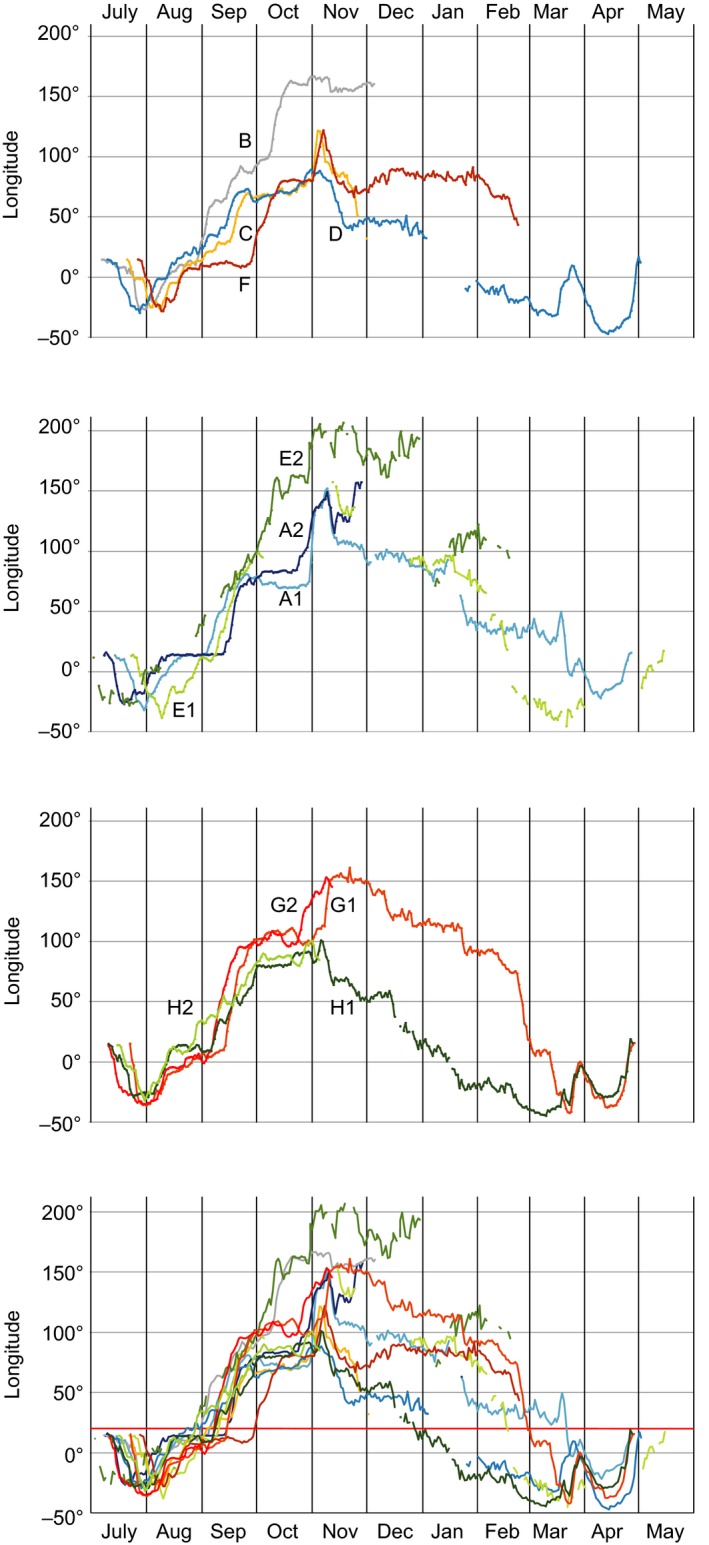
Longitude (degrees East) in relation to date during the annual migration cycle of arctic terns (from departure from breeding area until spring arrival in breeding area). Daily estimates of longitude are based on geolocator data for 12 journeys by eight individuals (cf. Table 1). Breeding longitude was 14.5E. The longitude of 20E (southern tip of Africa) is indicated by red line in the bottom graph. Negative and positive values refer to longitudes west and east of the Greenwich (0°) meridian, respectively, and longitudes plotted as 180–210E correspond to 180–150W. The upper graph shows the longitude data for the four individuals (inds. B, C, D, and F) tracked during a single journey. The second and third graphs show data for individuals tracked during two successive journeys (inds. A and E in second graph and inds. G and H in third graph), while longitude data for all journeys are plotted in the bottom graph (cf. Table 1). In these graphs, westerly longitudes have been plotted as negative, meaning that negative slopes of longitude change with time reflect westerly movement, while positive slopes reflect easterly movement

Estimated locations (lat/long data) are plotted (excluding equinox periods with invalid latitude estimates) for all journeys combined on the map in Figure 1 and on maps for each individual journey in Appendix 1. The longitudinal results for all 12 annual journeys are presented in Figure 2. Approximate dates, longitudes, and locations have been summarized in Table 1 for significant events in the annual cycle (derived primarily from the longitude data illustrated in Figure 2).

**Table 1:** Approximate dates and longitudes of Arctic tern migration (based on geolocator data from 12 journeys by eight individuals)

Ind	A1	A2	B	C	D	E1	E2	F	G1	G2	H1	H2	All		
													Range	Mean (*SD*)	*N*
Year	2008	2009	2008	2009	2009	2011	2012	2011	2013	2014	2014	2015			
Departure breeding area	13 July	9 July	7 July	21 July	11 July	24 July	beg June	28 July	21 July	9 July	10 July	15 July	7–28 July	15 July (7.1)	11
N Atlantic furthest W location	49N, 32W	50N, 27W	48N, 27W (56N, 26W)	19N, 27W (50N, 25W)	45N, 30W	50N, 38W	31N, 28W (56N, 26W)	49N, 28W	45N, 36W	39N, 35W	39N, 30W	48N, 32W	19−50N, 27−38W	43N, 31W	12
N Atlantic furthest W	28 July	18 July	29 July (26 July)	8 August (2 August)	27 July	8 August	20 July (9 July)	9 August	30 July	30 July	30 July	29 July	18 July to 9 August	29 July (6.9)	12
Passing equator	13 August	6 August	13 August	20 August	12 August	28 August	c. 30 July	29 August	9 August	8 August	10 August	8 August	30 July to 29 August	12 August (8.8)	12
Passing 20E into Indian Ocean	2 September	12 September	28 August	2 September	21–29 August	8 September	Mid‐August	28 September	13 September	5 September	6 September	26 August	26 August to 28 September	6 September (9.3)	11
Passing 50S to Antarctica	29 October	8 November (22 November)	8 November	2 November	27 October	beg October to beg November	c. 29 October	6 November	31 October	4 November	4 November	25 October	25 October to 8 November	1 November (4.8)	11
Furthest E	7 November	27 November	29 October	3 November	30 October	11 November	16 November	6 November	15 November	7 November	4 November	30 October	29 October to 27 November	7 November (8.5)	12
Furthest E, long	152E	158E	167E	122E	89E	158E	207E (153W)	122E	157E	153E	101E	101E	89−207E	141E	12
Antarctica, furthest W	11 March				13 March	13 March			22 March		9 March		9–22 March	13 March (5.0)	5
Antarctica, furthest W, long	23E				32W	40W			42W		45W		45W−23E	27W	5
Departure Antarctica	Mid‐March				16 March	Mid‐March			23 March		Mid‐end March		Mid‐March		5
N Atlantic furthest W location	33N, 22W				40N, 47W				27N, 38W		36N, 30W		27−40N, 22−47W	34N, 34W	4
N Atlantic furthest W	8 April				13 April				12 April		10 April		8–13 April	10 April (2.2)	4
Arrival breeding area	24 April				30 April				26 April		26 April		24–30 April	26 April (2.5)	4

Note

For the locations furthest to the west in the North Atlantic, approximate estimates of latitudes are given in addition to the longitude estimates (for both autumn and spring migration). Ranges, means, and samples sizes (no. of journeys) are given in the three right‐hand columns in bold. Standard deviations (in days) are given in addition to the mean dates for the selected events in the annual cycle of the terns. A2: temporary exit from Antarctica to 45S, 130E 15–22 November, return to S of Australia. No data after 27 November. B: similar westerly locations in the North Atlantic were recorded at different latitudes as indicated (autumn migration), no data after 3 December. C: similar westerly locations in the North Atlantic were recorded at different latitudes as indicated (autumn migration), no data after 30 November. D: stationary at about 35S, 20E 21–29 August, no data 3–23 January. E1: no data 4 October–10 November, 23 November–23 December, and 31 March–30 April. E2: fragmentary data June, similar westerly locations in the North Atlantic were recorded at different latitudes as indicated (autumn migration), no data 7–25 August, 1–8 September, and after 16 February. F: no data after 22 February. G2: no data after 10 November. H2: no data after 3 November.

Durations, distances, and travel speeds are schematically estimated for the main segments of the terns' annual migration cycle in Table 2. The terns completed an annual journey of about 50,000 km (45,000–60,000 km) with the fastest movements (450–500 km/day) during the autumn segment from the North Atlantic to the equator and the spring segment from Antarctic waters to the North Atlantic. During their travel segments off the African southwest coast (Benguela Current) and in the Indian Ocean (sometimes including Australian waters and the Tasman Sea), their speeds were clearly slower (about 175 km/day), indicating that flight transport was intermixed with periods with more stationary foraging. The slowest mean speed (about 60 km/day) occurred during the 4‐month period in the Antarctic zone, when molt and much foraging presumably occurred in the pack‐ice belt.

**Table 2:** Durations, distances, and travel speeds for different segments of the annual migration circuit of arctic terns

Segment	Mean dates	Duration (days)	Mean distance (km)	Min‐max distance (km)	Mean speed (km/day)
Breeding area – N Atlantic	15 July to 29 July	14	3,560		254
N Atlantic – Equator	29 July to 12 August	14	7,110		508
Equator – South Africa	12 August to 6 September	25	4,290		172
South Africa – Antarctica	6 September to 7 November	62	10,880	7,750–16,400	175
Antarctica, E to W	7 November to 13 March	126	7,890	5,450–10,990	63
Antarctica – N Atlantic	13 March to 10 April	28	12,710		454
N Atlantic – breeding area	10 April to 26 April	16	4,460		279
Total nonbreeding migration circuit	15 July to 26 April	285	50,900	45,330–59,520	179

Note

Durations are estimated from mean dates given in Table 1. Mean distances have been schematically calculated along loxodromes between mean locations as given in note below table (see also Table 1). Distances have also been estimated for the two journeys with shortest (ind. D) and longest (ind. E2) segments in the Indian Ocean and Antarctica (Table 1). Locations used for schematic distance calculations: Breeding area – N Atlantic (56N, 14.5E–43N, 31W), N Atlantic – Equator (43N, 31W–0N, 13W–0N, 5E), Equator – S Africa (0N, 5E–36S, 20E), S Africa – Antarctica (36S, 20E–40S, 100E–65S, 141E), Antarctica (65S, 141E–65S, 27W), Antarctica – N Atlantic (65S, 27W–30S, 5E–34N, 34W), and N Atlantic – breeding area (34N, 34W–56N, 14.5E). Locations for minimum distance: S Africa – Antarctica (36S, 20E–40S, 75E–65S, 89E), Antarctica (65S, 89E–65S, 27W). Locations for maximum distance: S Africa – Antarctica (36S, 20E–40S, 160E–65S, 207E), and Antarctica (65S, 207E–65S, 27W).

### 3.1 Autumn migration

After breeding (at about 56N, 14.5E), the terns departed on westerly courses to reach longitudes 27W–38W in the North Atlantic Ocean (at latitudes 19–50N, occasionally further north). In one case (ind E2), the tern did not return to its breeding site but roamed the North Atlantic during the summer before departing on its next annual journey. The sharply defined dip in longitudes indicated that the terns changed courses from westerly to southeasterly in the North Atlantic Ocean without stopping for any longer periods. By mid‐August (mean 12 August), the terns crossed the equator, and the records in the second half of August about longitude 10E reflect their southward movement in the Benguela Current off the southwestern African coast. This period in the Benguela Current ended when the terns changed course toward the east and passed south of Cape Agulhas (the southernmost tip of Africa) moving into the Indian Ocean (the longitude of 20E is marked in red in the bottom graph of Figure 2). This passage typically took place in the final days of August or the first days of September (mean 6 September), but in some cases the terns stayed for longer periods in the Benguela Current, not crossing 20E until 12 September (ind A2), 13 September (G1), or 28 September (F).

**Figure 3:**
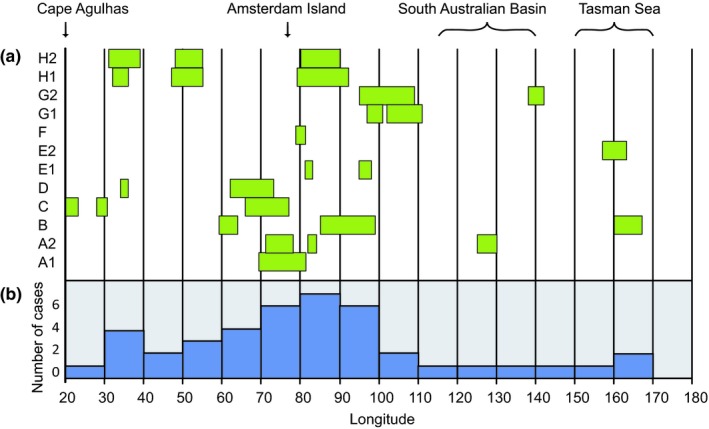
Longitude intervals (degrees east) where stopover occurred for arctic terns during their eastward migration across the Indian Ocean from South Africa (20E). Stopover was indicated by periods of interrupted and/or slow eastward change in longitude (≤1.0 degrees of longitude per day) based on inspection of longitude data (cf. Figure 2). Stopover periods lasted 5–44 days (on average 16 days, *n* = 26) and were associated with a mean eastward movement of 0.50 degrees per day (*n* = 26; range 0.11–1.0 degree/day). The upper graph shows longitude intervals of stopover for the different individuals and journeys (along the *y*‐axis, cf. Table 1). The number of stopover occasions recorded within different 10‐degree longitude intervals is summed in the lower graph. Estimated latitudes were in the range 24–42S (average 37S, *n* = 15), when available for these stopover periods

From the crossing of 20E and until reaching Antarctic waters south of 50S, the terns spent almost 2 months (September and October) traveling eastward in the Indian Ocean (and further east) with interludes of slow movement or stopover periods. Longitude intervals for these occasions of slow movement/stopover are given in Figure 3. Most of these occasions took place between 60E and 100E, within the latitude belt 35–45S, which is an oceanic area surrounding the Amsterdam Island in the southern Indian Ocean. Some periods of slow movement/stopover took place at localities far to the east after the terns had crossed the entire Indian Ocean, in the South Australian Basin (inds A2 and G2) and the Tasman sea (inds B and E2), respectively (Figure 3).

A rather synchronized event during the autumn migration was the terns' movement across 50S into the Antarctic zone, which took place in the period 25 October–8 November (mean 1 November with a standard deviation of 4.8 days; Table 1). Longitude data were available for 12 such crossings of the Antarctic Convergence zone (at about 50S), and in 10 of them, there was a rapid increase in longitude, indicating that the terns made a fast eastward movement during the crossing. In most of these cases, there was a compensatory westward movement immediately after arrival in the Antarctic waters, as reflected by a distinct peak in longitude in association with the crossing (Figure 2). For the 10 crossings with eastward movement, the eastward displacement ranged between 10 and 55 longitude degrees (mean 27 degrees), corresponding to eastward distances in the range 700–4,000 km (mean about 2,000 km; distance = longitude degrees × 60 nautical miles × cos [lat]).

Two crossings of the Antarctic Convergence were exceptional in the sense that they were not associated with eastward displacements: Ind B moved 15 longitude degrees to the west during the crossing and ind G1 crossed due south with little change in longitude.

In a few cases, the terns did not reverse to a westerly course immediately after arriving in the Antarctic zone but they continued eastward during a week or two before changing to westward movements, thus not reaching their furthest east until well after their crossing of the Antarctic Convergence (ind A1, G1; Table 1). On its second annual circuit, individual E (track E2) spent about 3 weeks in the Tasman Sea at longitudes 140–165E, from where it crossed into Antarctic waters and at the same time moved rapidly eastward to longitude 200E (= 160W) which is in the Ross Sea. This individual stayed in the Ross Sea region until the end of December when it made a rapid westerly leap (Figures 1 and 2).

### 3.2 Period in the Antarctic zone

During the terns' period in the Antarctic zone, from November until mid‐March, estimated latitudes were in the range 60–70S. This indicates that the terns most probably were staying in the pack‐ice zone. However, the uncertainty in the provisional and biased latitude estimates during the Antarctic summer makes it impossible to tell whether they were at the edge or in the inner parts of this zone.

**Figure 4:**
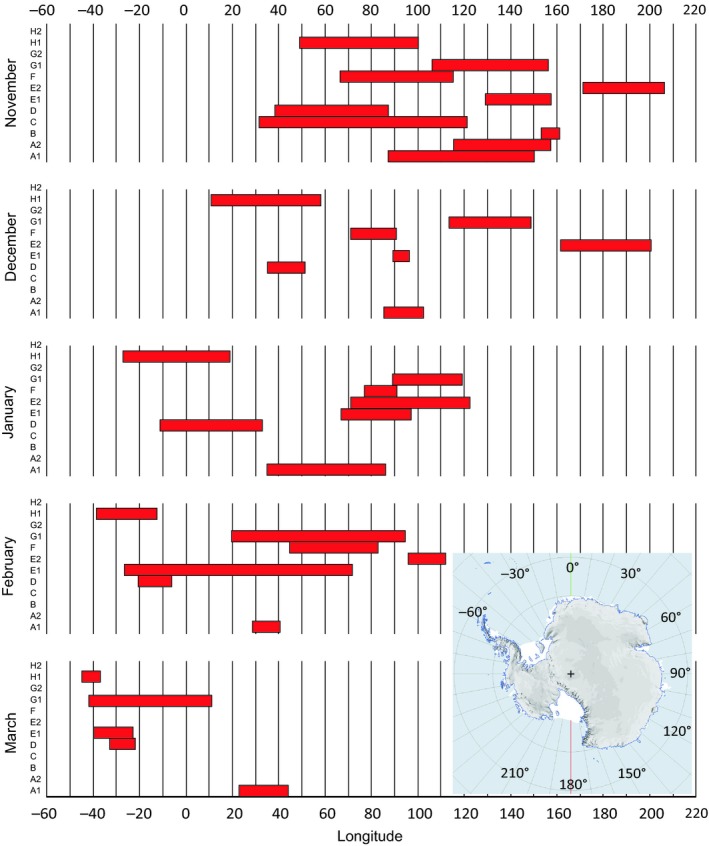
Longitude ranges of arctic terns in Antarctic waters (south of 50N) during different months of their wintering period November to March (based on geolocator data for 12 journeys by eight individuals). Individuals and journeys are indicated on the *y*‐axes of the monthly graphs (cf. Table 1). Inset map shows the longitudinal range from −60 (= 60W) to 180E and further to 210E (corresponding to 150W) as plotted on the *x*‐axis

**Figure 5:**
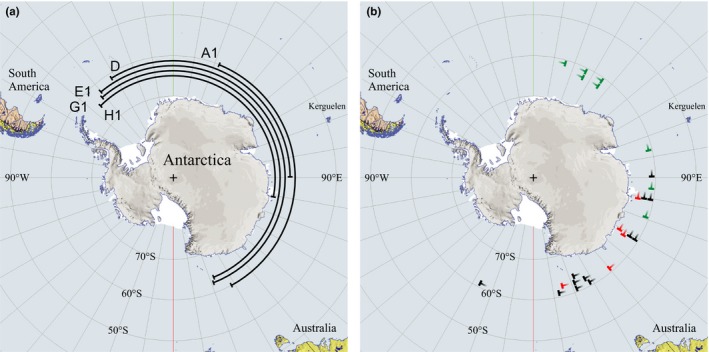
(a) Total longitudinal ranges from furthest east to furthest west for five terns during their stay in the Antarctic zone. Individuals and journeys are indicated according to Table 1. (b) Longitudes of furthest east in the Antarctic zone as recorded for arctic terns from different populations (based on geolocator data). Black symbols refer to terns from breeding sites in Sweden (this study, *n* = 12, Table 1), red symbols to terns from the Netherlands (from Figure 1 in Fijn et al., 2013; *n* = 5), and green symbols to terns from Greenland/Iceland (from Figure 1 in Egevang et al., 2010, *n* = 11 with three terns not moving east of longitude 0⁰ excluded from plotting because furthest east could not be read from figure)

The terns reached their easternmost longitudes at or soon after their arrival in the Antarctic zone, and after this, they generally moved westward during the following months in the Antarctic zone. The resulting westward change in distribution during the Antarctic summer months is clearly seen both on the individual and on the population level (Figure 4). While the mean longitude when the terns were furthest to the east was 141E (7 November; *n* = 12 journeys; Table 1), the mean longitude on 1 January was 71E (range 19E–114E; *n* = 6) and it was 27W (which is in the Weddell Sea; *n* = 5) when the terns reached furthest west in the Antarctic zone about 13 March (Table 1). The total longitudinal range from furthest east to furthest west was available for five terns (ranges between 121 and 199 degrees; Figure 5a).

### 3.3 Spring migration

Departure from the Antarctic zone took place in mid‐March, and longitude data indicated a northeasterly course during the terns' northward crossing of the Antarctic Convergence (Figure 2). After reaching a longitude peak in the last days of March (when the terns presumably were far off the southwestern African coast at longitude 0–5E), the longitudes became more westerly again in April when the terns moved toward northwest up the open Atlantic Ocean further offshore from Africa compared with autumn migration. The longitude peak in the last days of March was most prominent for ind D, G1, and H1 (cf. Figure 2), indicating an eastward displacement across about forty degrees of longitude during the terns' northward crossing of the Antarctic Convergence, while this pattern was less clear for ind A1 and E1 (Figure 2). In contrast to the other four individuals providing data about the spring departure, individual A1 did not reach the Weddell Sea, but its furthest west was 23E on 11 March. Of course, without valid latitude estimates the interpretation of the departure routes based on only longitude data remains speculative. Like in autumn, the terns showed a well‐defined “longitudinal dip” of furthest west in the North Atlantic at longitudes 22–47W (latitudes 27–40N, *n* = 4; Table 1) in April, before they headed eastward back to their breeding sites (Figures 1 and 2).

### 3.4 Different populations

#### 3.4.1 Autumn migration

The migration patterns and annual cycles of arctic terns from the Baltic Sea in southern Sweden, as analyzed in this study, may be compared with corresponding results for breeding populations in Greenland (geolocator data for 11 individuals, one from Iceland; Egevang et al., 2010), the Netherlands (*n* = 5; Fijn et al., 2013), and Alaska (*n* = 6; Duffy, McKnight, & Irons, 2013; McKnight et al., 2013). While Greenlandic, Dutch, and Baltic terns followed the East Atlantic flyway toward the Antarctic winter range, the terns from Alaska followed the second main flyway in the East Pacific Ocean. In addition, there are recent geolocator data about departure and arrival timing of British arctic terns (*n* = 47; Redfern & Bevan, 2019).

The Baltic and Dutch terns (breeding sites were only about 600 km apart) showed similar migration patterns and schedules. However, there were striking differences between these two populations and the Greenlandic birds. This was unexpected since one may have assumed, as did Salomonsen (1967), that all arctic terns in the East Atlantic flyway would show similar migration and wintering habits. Clear indications of important differences in Antarctic nonbreeding ranges between terns from Greenland and the Netherlands, suggesting a significant degree of migratory connectivity, were pointed out by Fijn et al. (2013).

After flying round the southern cape of Africa, the Baltic and Dutch terns traveled eastward (with interludes of staging/stopover) across the Indian Ocean to reach furthest east when crossing into Antarctic waters in the longitude interval of 89–207E (mean = 141E, *n* = 12 journeys; Table 1) for the Baltic birds and 102–165E (mean = 123E, *N* = 5; Fijn et al., 2013) for the Dutch birds (Figure 5b). A few terns from both populations reached as far east as the Tasman Sea. One of the Baltic terns crossed from the Tasman Sea to Ross Sea (at 207E = 153W) in the Antarctic region. Such easterly movements in the Southern Hemisphere were much less prominent among the Greenland birds. Four of them did not move east of the southern tip of Africa (20E) at all but headed directly for the Weddell Sea region. Seven of them reached furthest east in the longitude interval 27–109E, none of them reaching as far east as Australia. For the Greenlandic birds, median longitude furthest to the east was 27E (*n* = 11; Egevang et al., 2010) which is highly significantly different from the combined data of Baltic and Dutch terns (Median test, Fisher exact probability *p* = .001, two‐tailed).

There was a tendency for the Baltic birds to have a slightly advanced migration schedule compared with the Dutch (and British) birds, with mean (Baltic birds) or median (Dutch) dates as follows: departure from breeding regions 15 July (Baltic) versus 27 July (Dutch), passing equator/arrival at Benguela Current 12 August (Baltic) versus 23 August (Dutch), passing 20E/leaving Benguela Current 6 September (Baltic) versus 25 September (Dutch), and arrival in Antarctic waters 1 November (Baltic) versus 12 November (Dutch) (Table 1, Fijn et al., 2013). Median departure dates for arctic terns breeding on the North Sea coast of Britain were 25 July and 4 August in two different years (Redfern & Bevan, 2019). These comparisons of the annual temporal cycle must be regarded as provisional, based on small sample sizes and approximate timing estimates from different years.

The autumn migration schedule was much later for the Greenland terns. They arrived at the North Atlantic staging area on 22 August (mean date; Egevang et al., 2010), when Baltic and Dutch terns had already passed the equator and arrived in the Benguela Current. The Greenland terns made a surprisingly long stop in the North Atlantic, on average 25 days (Egevang et al., 2010), in comparison with the Baltic terns that passed this area in slow movement without a well‐defined stop and the Dutch birds with only a brief stop of 6 days on average (Fijn et al., 2013). When the Greenland terns finally departed from the North Atlantic, on average on 15 September (Egevang et al., 2010), the Baltic terns had already rounded Africa and traveled into the Indian Ocean and the Dutch terns were in the process of doing so.

These comparisons show that autumn migration was organized in clearly different ways between the Baltic/Dutch birds on one hand and the Greenland terns on the other hand. The total duration of autumn migration was about 3.5 months for all three populations. The Baltic and Dutch birds spent 1.5–2 months of this time in the eastward migration segment (interspersed with staging) across the Indian Ocean (eastward to the Tasman Sea). However, the Indian Ocean segment was lacking or of very limited importance among the Greenland terns. Rather, they exploited staging regions in the North Atlantic and along the African coast (Benguela Current) much longer than the Baltic and Dutch birds did. Four of the Greenland birds migrated southward along the Brazilian coast, spending a staging period off Argentina (possibly partly overlapping with terns from Alaska staging at the Patagonian Shelf; see below), before proceeding toward the Weddell Sea. The autumn migration of arctic terns from Alaska also lasted about 3.5 months (from about 1 August to mid‐November; McKnight et al., 2013), when the terns exploited staging areas in the California Current and the northern and southern Humboldt Current along their East Pacific flyway (McKnight et al., 2013). Five of the six terns migrated across at least 800 km of South American land (from the Pacific to the Atlantic Ocean across the Andes and Patagonia) to stage at the Patagonian Shelf before continuing to the Weddell Sea region (Duffy et al., 2013; McKnight et al., 2013).

#### 3.4.2 Wintering and spring migration

The arctic terns were distributed over very wide longitudinal sectors during the wintering period in the Antarctic pack‐ice zone. The widest range was recorded for the Baltic population, spanning 240 degrees of longitude, which corresponds to a distance of about 13,000 km along the 60S parallel. This very wide distribution was due to differences between individuals, but most importantly to the westward movements of individuals during the course of the wintering period. Such individual movements extended over longitudinal sectors of 121–199 degrees (6,700–11,000 km along 60S) for five individuals with data for both furthest east and west (Figure 5a). Since individuals were widely scattered at a given time, the population range during a given month was also wide, often about 150 degrees of longitude for the Baltic population (Figure 4).

Individual movements during the wintering period have not been analyzed for the Dutch, Greenland, and Alaska populations. Comparisons between populations must therefore be limited to the population‐specific wintering ranges. The main longitude range for the Dutch population was 80–150E (Fijn et al., 2013), thus overlapping with the Baltic terns. In contrast, Greenland birds had a more westerly main wintering range, in the longitudinal sector 60W–30E (primarily the Weddell Sea region) with a few individuals arriving in the pack‐ice zone at more easterly longitudes (80–110E) in the beginning of the wintering period (Egevang et al., 2010). Among the terns from Alaska, one individual traveled west of the Antarctic Peninsula to about 140W (at the boundary of Amundsen and Ross Seas), while the other five traveled to the Weddell Sea region (10–45W; McKnight et al., 2013). This means that the total wintering sector spanned about 130 degrees of longitude and that the majority of Alaska terns overlapped in distribution with the Greenland birds.

The period in the Antarctic zone lasted 4.5 months for the Greenland, Dutch, and Baltic birds (no data for spring departure and migration of the Alaska birds), with the Baltic and Dutch terns departing northward in mid‐March and the Greenland birds a month later. While the Greenland birds departed from the sector 0–30W in the Weddell Sea region, the Dutch birds seemed to depart from areas a bit further east, in the sector 0–30E (Egevang et al., 2010; Fijn et al., 2013). Spring departure was recorded for five Baltic terns, with four of them starting from the Weddell Sea region (32–45W) and the fifth from a more easterly longitude (23E; Table 1). The terns from these three populations followed very similar spring routes up the Atlantic Ocean further offshore from the continents than during autumn migration. The spring journeys from Antarctic waters to breeding sites were remarkably fast, lasting only about 40 days among the terns from all three populations.

## 4 DISCUSSION

We are now in a position to evaluate the key assumptions and predictions put forward by Salomonsen (1967) about the migration system of the arctic tern (cf. Introduction1). There are many aspects of the early reconstructions of the arctic tern migration system that are verified by the new geolocator data. It stands out as a remarkable and impressive achievement by Salomonsen (1967; and also Storr, 1958) to provide such a useful hypothetical synthesis. This was built on an insightful and scholarly interpretation of sparse and scattered field observations and ringing recoveries (building on earlier syntheses) in relation to environmental (wind patterns, low‐pressure trajectories, and pack‐ice distribution) and behavioral (flight performance) conditions. This shows that early studies of bird migration, based on field/ringing data, are certainly worth considering carefully when analyzing results from modern tracking techniques. However, far from all of Salomonsen's ideas stand the test of the new tracking results. Such falsification paves the way for the development of a new view on the arctic tern migration system.

### 4.1 Southward migration to the pack‐ice zone and the segregation between populations (aspects [a] and [e] in Introduction)

**Figure 6:**
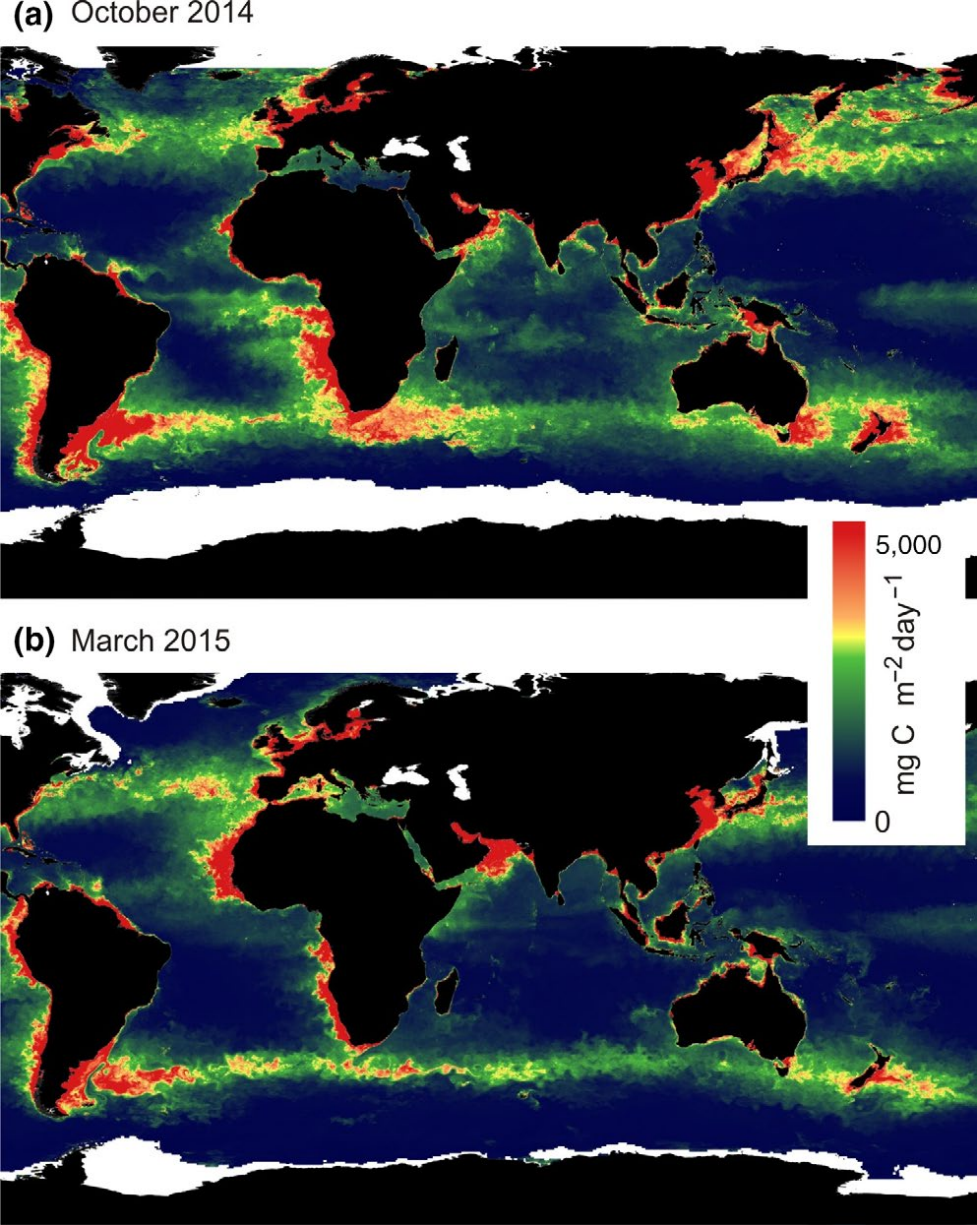
Geographical distribution of marine productivity (monthly net primary production measured as mg Carbon per m^2^ and day; see Behrenfeld & Falkowski, 1997) during October (a) and March (b). Monthly ocean productivity data were downloaded from Oregon State University Ocean Productivity Site (www.science.oregonstate.edu/ocean.productivity/index.php) for October 2014 and March 2015 (selected as example years; the monthly geographical patterns are very similar between years). During October, Baltic terns traveled eastward while exploiting the productivity zone extending from the waters at South Africa across the Indian Ocean to the Tasman Sea. In the end of March, the terns moved very rapidly, presumably without much foraging, from the Weddell Sea region in Antarctica to the North Atlantic productivity zone

Salomonsen's (1967) suggestion of an important eastward autumn migration across the Indian Ocean, with some terns passing also Australian and New Zealand waters, is supported in an impressive way by the tracking data for the Baltic and Dutch populations. But the main explanatory factors suggested by Salomonsen (1967) and Storr (1958) for such an eastward migration segment (drift by strong westerly winds, dependence on low‐pressure trajectories, and poor flight performance due to exhausted condition and worn flight feathers) seem to be unlikely when considering the fact that the new geolocator data revealed different behavior among different tern populations following the East Atlantic flyway. The fact that terns from Greenland often forwent any longer eastward migration trip into the Indian Ocean but instead crossed more directly to the Antarctic pack‐ice zone strongly suggests that the terns are readily capable of directly crossing the Antarctic Convergence. Rather, the ultimate explanation for the transverse migration across the Indian Ocean is most probably a high availability of food making these marine regions favorable for the terns to exploit prior to the time when it is optimal for them to proceed to the Antarctic pack‐ice zone. This is nicely supported by ocean productivity data showing the rich waters in September–November in the latitude belt 30–45S (along the Subtropical Convergence zone) extending from southern Africa across the Indian Ocean, off southern Australia, and into the Tasman Sea (Figure 6). These marine regions seem to offer similarly favorable staging possibilities for the terns as other marine productivity hotspots, such as the California and Humboldt Currents and the Patagonian shelf that are exploited by the Alaska terns on their autumn migration ((McKnight et al., 2013). The North Atlantic and Benguela Current are used in a corresponding way by the terns in the Atlantic flyway. McKnight et al. (2013) described such exploitation of marine high‐production regions along the route as a stepping‐stone migration pattern. These stepping stones serve as temporary living stations in the annual cycle of the terns.

One may assume that the Antarctic pack‐ice zone is not becoming useful to the terns until about 1 November, which is springtime in Antarctica. This means that the pelagic living stations exploited by the different tern populations may be regarded, not only as an exploitation of a marine seasonal resource peak but also as a stay awaiting the development of beneficial spring conditions in the Antarctic pack‐ice zone. This is analogous to the cases of itinerancy with several nonbreeding living stations among long‐distance songbird migrants (Curry‐Lindahl, 1981; Pearson, 1990; Thorup et al., 2017). One example is the red‐backed shrike staying for 2 months in autumn in the Sahel zone exploiting the peak of resources associated with the rainy season north of the equator and at the same time waiting for the rainy season to start in southern Africa, allowing the shrikes to arrive there in November–December (Pearson, 1990; Tøttrup et al., 2012).

Why is the Indian Ocean/Tasman Sea exploited mainly by the terns from northwest Europe and not much by Greenland terns? Geographical segregation in migration patterns may develop as a result of asymmetric competition and differential migration costs among populations (Lundberg & Alerstam, 1986). With their earlier breeding period, terns from Northwest Europe can exploit the Indian Ocean/Tasman Sea belt for a long period (2 months), avoiding competition with later‐breeding populations in the Atlantic Ocean. In addition, they may benefit from competitive release also at their more easterly wintering regions in the Antarctic pack‐ice zone. However, the extra‐long migration distances may add to the cost side. For the later‐breeding terns from Greenland, the cost–benefit balance will be different. They may stay long in the North Atlantic and Benguela Current with little overlap and competition with European tern populations, and their migration costs may be mitigated. Future detailed migration data for more tern populations will allow critical comparisons of the spatial and temporal population overlaps during the annual cycles. Such comparison may be the key to understand the evolution of geographical segregation (connectivity patterns) between populations in the arctic tern global migration system.

Even if the dominance of strong westerly winds in the Indian Ocean/Tasman Sea belt is not the ultimate explanation for the terns' movements here, it is most probably a necessary contributory cause. The terns can get access to this productive marine belt by traveling with assisting winds from southern Africa. The suggestion that the terns adopt a flight strategy adapted to the low pressure trajectories to facilitate efficient crossing into Antarctic waters (Salomonsen, 1967) gains some support from the longitudinal peaks (Figure 2), showing rapid eastward followed by westward movements when the terns crossed 50S and arrived in Antarctic waters. Such a movement pattern was recorded for the majority (but not all) of the southward crossings of the stormy waters of the Antarctic Convergence zone. With the clockwise circulation around low pressures in the Southern Hemisphere, this may indicate that the terns start the southward crossing in northwesterly winds from an approaching low‐pressure system, later to encounter easterly winds south of the passing low pressure systems. Also after spring departure from the Weddell Sea did most of the terns show a distinct “local longitude peak” indicating temporary eastward displacement during the northward crossing of the Antarctic Convergence toward the Southern Atlantic Ocean. With more high‐resolution GPS tracking data becoming available in the future, it will also be possible to investigate more in detail how the terns manage the passing low‐pressure systems and the changing winds for their passage across these stormy oceans.

We think that there is little reason to suspect that the terns are dramatically affected by poor flight performance (worn flight feathers) and a more exhausted condition during autumn (pre molt) compared with spring (post molt) migration. Worn flight feathers are no doubt associated with significantly reduced flight efficiency as indicated in a few studies (Chai & Dudley, 1999; Hedenström, 2003; Williams & Swaddle, 2003), but the detrimental effect is probably not huge. The adult terns seem to have diverse behaviors (depending on population) in their autumn migration in the Southern Hemisphere. This includes extensive migration in the Indian Ocean/Tasman Sea belt (Baltic and Dutch terns) and overland flights in South America (Alaska terns; Duffy et al., 2013) that cannot easily be assigned to a markedly poor flight performance. In addition, annual survival rate is very high (close to 90%; Cramp, 1985), indicating that the terns are in more control of the situation than suggested by Salomonsen (1967) and Storr (1958).

### 4.2 Stay in the pack ice and spring departure (aspects [b] and [c] in Introduction)

Salomonsen (1967) suggested that the arctic terns were subjected to eastward displacement after arriving at the edge of the pack‐ice zone, because of the prevailing westerly winds. Only after molt did they compensate for this by westward migration, when they could also benefit from the polar easterlies closer to the Antarctic coast (Salomonsen, 1967). However, our geolocator data did not confirm such a general pattern. Rather, the terns showed an overall westward movement from November until the beginning of March, although with temporary exceptions of eastward movement or constant longitude (Figures 1, 2, 4 and 5). An accelerated westward movement was observed in some cases in January and February (Figure 4), giving at least partial support to Salomonsen's predictions.

**Figure 7:**
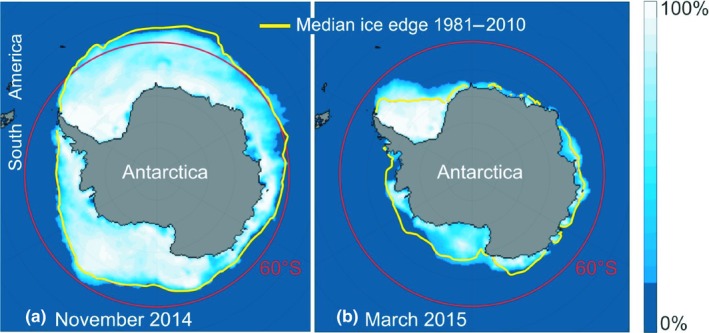
Extension of the Antarctic pack‐ice zone in November (a) and March (b), which are the times for the arrival of arctic terns at this zone (November) and departure from it (March; cf Table 1). The illustration was downloaded from the National Snow & Ice Data Center (https://nsidc.org/data/seaice_index/archives/). November 2014 and March 2015 were selected as example years. The monthly patterns are similar between years as seen from the median distributions 1981–2010, indicated by yellow lines). During their four‐and‐a‐half months stay in the Antarctic pack‐ice zone, the terns moved westward toward the Weddell Sea region (Figures 4 and 5) where much sea ice still remains in March

There was also some support for the suggestion that the Weddell Sea represents the main region of exodus from Antarctica at the initiation of the terns' spring migration (although spring departures from more easterly longitudes were also indicated; Fijn et al., 2013). Still, it seems uncertain whether the main reason for this is a precompensation for the expected eastward drift when crossing the zone of strong westerlies on the way up into the Atlantic Ocean (as suggested by Salomonsen, 1967), or whether the Weddell Sea offers particularly favorable foraging conditions during the Antarctic autumn when the pack‐ice distribution is at its minimum. At this time, much pack ice still remains in the Weddell Sea region (Figure 7), possibly offering optimal foraging conditions for premigratory fattening. In spring, the terns reach their distant breeding quarters by a travel period of only about 40 days.

### 4.3 Circumpolar movements (aspect [d] in Introduction)

Salomonsen (1967) predicted that adult terns, even if reaching the pack‐ice zone at far easterly longitudes, would migrate back toward the west rather than continuing eastward to complete a circumpolar movement during their period in the Antarctic zone. This was verified by the geolocator results for the Greenland, Dutch, and Baltic terns (Egevang et al., 2010; Fijn et al., 2013; this study). Even the Baltic tern (ind. E2) moving as far east as the Ross Sea after its staging period in the Tasman Sea migrated back westward rather than completing a circumpolar migration which would have been the shortest distance back to the Atlantic Ocean.

However, for young terns often spending not only their first winter but also their first summer and succeeding winter in the Southern Hemisphere, Salomonsen (1967) suggested that circumpolar migration (completed during 1 or 2 years) may be of more regular occurrence. Since no tracking data are available for young terns, this prediction remains to be tested. There are a number of reasons to expect that circumpolar movements do in fact occur, not only among young terns but occasionally also among adults. The observations of thousands of arctic terns apparently passing northward and eastward across the Antarctic Peninsula into the Weddell Sea in the beginning of March provide a strong indication of terns arriving into the Weddell Sea not only from the east but also from the west at the time of spring departure (Gudmundsson, Alerstam, & Larsson, 1992). Satellite tracking has demonstrated the regular occurrence of circumpolar migration among other species of seabirds in the Southern Ocean (e.g., Croxall, Silk, Phillips, Afanasyev, & Briggs, 2005, Weimerskirch et al., 2014). Some remarkable ringing recoveries suggest exceptional movements also by adult arctic terns, for example, a 10‐year‐old tern from Greenland that was shot in the Colombian Andes at 2,000 m asl on 16 June (Alerstam, 1985) and another 10‐year‐old tern from the Baltic Sea that was recovered close to the Andes in Bolivia on 8 April (Fransson in litt.; https://birdringing.bioatlas.se/). A speculative interpretation for both these terns is that, in spite of their age and experience, they have temporarily joined terns migrating northward in the East Pacific flyway after near‐circumpolar migration in the Antarctic zone, trying to complete the circumpolar movement back into the Atlantic by crossing the South American continent.

## 5 CONCLUDING REMARK

The global migration system of the arctic tern offers an extraordinary possibility to understand the adaptive values and constraints in pelagic life cycles, as determined by environmental conditions (marine productivity, wind patterns, low‐pressure trajectories, pack‐ice distribution), inherent factors (flight performance, molt, flocking), and effects of predation/piracy and competition. In this study, we have tested a number of hypotheses put forward by Salomonsen (1967) before the time of the modern tracking revolution. A number of these predictions have stood the tests so far, and others have been falsified or modified. A fascinating story is unfolding about the evolutionary ecology of the world's migration champion.

## CONFLICT OF INTEREST

None declared.

## AUTHOR CONTRIBUTIONS

TA, JG, PO, and RS planned the study; JG organized the project and grant applications; PO and RS conducted fieldwork with help from JG; RS analyzed geolocator data with input from all authors; all authors interpreted the results; TA and RS prepared a draft manuscript; and JB and JG prepared illustrations. All authors revised, read, and approved the final manuscript.

## ETHICAL APPROVAL

The study was approved by the Lund University ethical committee (Malmö‐Lunds djurförsöksetiska nämnd; approvals M101‐07, M112‐09, M100‐12, and M56‐15).

## Data Availability

All geolocator data included in this study and the processed results are freely available from the Dryad Repository. Light‐level data: available as text files, deposited at Dryad Repository (https://doi.org/10.5061/dryad.d6080nt). Location data: available as spreadsheet file, deposited at Dryad Repository (https://doi.org/10.5061/dryad.d6080nt).

## APPENDIX 1

Maps (Mercator projection) showing estimated locations of arctic terns from the Baltic Sea during their annual migration cycle. Daily locations based on geolocator data are presented on separate maps for each journey (12 maps for the 12 annual journeys by eight individuals). Individual and journey are indicated on each map, with summary data for the different individuals and journeys given in Table 1. Locations are missing due to uncertain and invalid latitude data during periods around the autumn and spring equinoxes (longitude data during equinox periods are presented in Figure 2) and during periods of midnight sun. Such gaps in locations are schematically indicated by arrows. Locations have been provisionally plotted during the terns' wintering period in the Antarctic in spite of the fact that latitude could not be properly estimated but only inferred to be south of 60S during the southern polar summer (see methods about latitude bias for these locations).
